# Anesthetic Considerations of Organ Procurement After Brain and Cardiac Death: A Narrative Review

**DOI:** 10.7759/cureus.40629

**Published:** 2023-06-19

**Authors:** Michael B Brown, Apolonia E Abramowicz, Peter J Panzica, Garret Weber

**Affiliations:** 1 School of Medicine, New York Medical College, Valhalla, USA; 2 Department of Anesthesiology, Westchester Medical Center, Valhalla, USA

**Keywords:** organ donation, organ donor management, organ procurement, cardiac death, brain death organ management

## Abstract

Organ donation procedures have become more frequent in the US as the need for transplants is increasing. Defining the anesthesiologist's role in organ donations after brain and cardiac death is important, as is understanding its ethics and practical physiologic and perioperative implications. Despite this, there are few papers specifically addressing the anesthetic management of organ donors. This review summarizes the preoperative, intraoperative, and postmortem considerations for the anesthesiologist involved in organ donation after either brain or cardiac death. A search of the published literature was performed using PubMed, Excerpta Medica dataBASE (EMBASE), and Google Scholar in March of 2022 for articles addressing anesthetic considerations of organ procurement surgeries after brain and cardiac death. This review demonstrates that anesthesiologists play a significant role in the organ procurement process. Their role in the perioperative management of the donor may affect the outcomes of organ transplantation. The gap between the number of organs harvested and the number of patients awaiting organ transplantation remains high despite continued efforts to increase the number of available organs. Perioperative management of organ donors aims at counteracting the associated unique physiologic derangements and targets optimization of oxygenation of the organs intended for procurement. Optimizing care after death can help ensure the viability of organs and the best outcomes for recipients. As organ donation after cardiac death (DCD) becomes more frequent in the US, anesthesiologists should be aware of the DCD classifications of donors and emerging novel perfusion techniques.

## Introduction and background

In January 2022, there were over 106,000 people in the US on transplant waiting lists [1]. Each year, over 8,000 people die waiting for organs [1,2]. Although there is an organ shortage, donations from deceased donors have doubled over the last 20 years [2]. There is a large body of literature on the intensivist management (ICU) of organ donors [3,4]. However, few papers provide guidance for the anesthetic management of organ donors, and adherence and implementation of these recommendations are unknown [5,6]. As organ procurement procedures are increasing, there is a call for greater involvement and oversight by anesthesiologists in organ procurement and recovery [5,6]. According to a single-center study by Lele et al., anesthesiologists manage an average of less than one brain-dead donor annually [7]. Although rare, the anesthesiologist’s involvement in organ procurement can affect the outcomes of organ recovery and transplantation [8]. For this reason, anesthesiologists should be familiar with the current definitions and policies governing organ donation and donor management goals [8,9]. This narrative review will present the current state of organ procurement in the US, the legal aspects of organ procurement, and the ethical considerations of the determination of death. We will also address preoperative, intraoperative, and postmortem considerations for the anesthesiologist involved in the organ donation process after brain and cardiac death. 

Methods

A search of the published literature was performed using PubMed, Excerpta Medica dataBASE (EMBASE), and Google Scholar in March of 2022 for articles addressing anesthetic considerations of organ procurement after brain and cardiac death. The full electronic search strategy can be found in Table 1. Articles that focus on organ procurement were chosen. Ninety-eight articles were identified in the initial search terms. Six articles were omitted from the search as they were not available in an English language version. References of all selected articles were also searched to identify additional sources. The search was not limited by the article type.

**Table 1:** Search Strategies EMBASE: Excerpta Medica dataBASE

Database	Search Strategies
PubMed	(organ donation OR "Tissue and Organ Harvesting"[Mesh]) AND ("Anesthesia"[Majr] OR "Anesthesiology"[Majr]) AND (brain death OR cardiac death)
EMBASE	'organ donation anesth' OR (('organ'/exp OR organ) AND donation AND anesth) AND organ AND procurement AND anesthesia
Google Scholar	“Anesthetic management in organ procurement after brain and cardiac death”

## Review

Donation after brain death and cardiac death

According to the American Academy of Neurology (AAN), brain death or death by neurologic criteria is defined as the irreversible cessation of the entire brain function [10]. A person who is declared brain dead and/or fits the criteria for brain death under the AAN, institutional guidelines, or state laws should be considered for organ donation. Donation after brain death (DBD) is the primary source of organs in the US [5,11]. In 2021, there were 9,674 organ donors who met the criteria for brain death [11].

**Figure 1:**
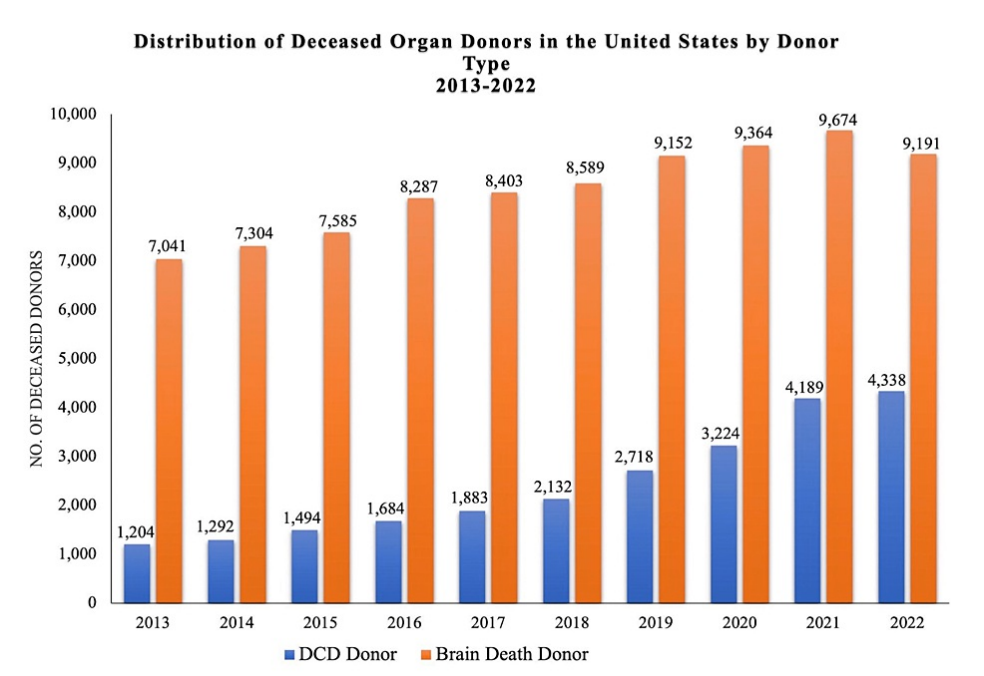
Distribution of Deceased Organ Donors in the United States by Donor Type, 2013-2022 Original figure made from data retrieved from Organ Procurement & Transplantation National Database on January 2, 2023, from https://optn.transplant.hrsa.gov/data/view-data-reports/national-data/. Citation: [6].

Cardiac death is defined as when a patient has irreversible cessation of circulatory and respiratory function [12]. After cardiac death, a patient is eligible to become an organ donor, which is known as donation after circulatory death (DCD). In 1995, the demand for organs greatly outpaced the number of DBD donors, and institutions sought to expand the donor pool. Subsequently, the Maastricht group from the Netherlands published their protocols for DCD [13]. Recognizing public hesitancy, in 1997, the US Institute of Medicine supported DCD and stated that it is medically and ethically acceptable in order to increase the available supply of organs for transplantation in the US [14]. Today, DCD accounts for about 10% of transplanted organs in the US [15]. The number of DCD donors has doubled since 2017. In 2017 and 2021, there were 1,883 and 4,189 DCD donors, respectively, (Figure 1).

Anesthesiologists are involved in DCD and should be well-versed in the legal, ethical, and medical considerations to facilitate the patient's best interest after death [6,16]. Most patients who are considered for DCD have end-stage cardiopulmonary disease, neuromuscular disease, and devastating irreversible neurologic injury, which requires life-sustaining treatment but does not meet all the criteria for brain death [6].

Legal and ethical aspects of organ procurement 

Organ Donation in the US

There are two general models that countries around the world use for organ donation. The “presumed consent model” legitimizes the public’s interest in deceased organ donation and allows the recovery of organs after death without prior authorization [17]. The other model of organ donation presumes individuals have ownership of their organs, which cannot be procured without consent or authorization. The US follows the latter model. It is referred to as the “donation model” [17-19]. It is regulated by the legal framework known as the Uniform Anatomical Gift Act (UAGA) [20].

The UAGA governs organ donation for the purpose of transplantation and medical research. It was adopted more than 50 years ago, just as the first human heart transplants were performed [20]. The UAGA and its revisions are geared toward increasing the organ donor pool. Under the UAGA, donations are considered a gift of generosity; organs cannot be used for financial gain [19,20]. Furthermore, the UAGA states that the next of kin can consent to organ donation. The latest revision of the UAGA from 2006 has three main goals: 1) to encourage the public to make organ donations, 2) to give every individual an opportunity to donate organs after meeting the criteria for either brain or cardiac death, and 3) to strictly adhere to an individual's wishes, including respecting the right to donate or not to donate organs [21].

Dead-Donor Rule and the Uniform Determination of Death Act

The ethical framework for organ donation in the US is known as the dead-donor rule [22]. It states that a patient’s life should not be terminated for organ donation, and they must be declared dead before any organs are retrieved [22,23]. Under the dead-donor rule, a uniform definition of death was needed, and in 1968, the Harvard Medical School Ad Hoc Committee on Donation after Brain Death sought to examine the definition of brain death [24]. It concluded that patients may be declared dead when they irreversibly lack (1) responsiveness, (2) spontaneous breathing, and (3) brainstem reflexes. This definition was applied for over a decade until advancements in modern life support technology made this definition of death insufficient [25-30]. In 1980, the National Conference of Commissioners on Uniform State Laws provided a stricter definition of death known as the Uniform Determination of Death Act (UDDA) in order to provide clarity on the determination of death in the age of advanced life support. The UDDA states that an individual is dead if they have sustained either irreversible cessation of circulatory or respiratory functions (without assistance from mechanical circulation or ventilation), known as cardiac death, or irreversible cessation of all functions of the entire brain, including the brain stem, known as brain death [31]. The UDDA is now accepted as a standard for declaring death in the US and most of the world [32]. Therefore, once the criteria for irreversible brain death or cardiac death are met, so is the legal threshold for seeking organ donation consent [31,32]. A summary of relevant definitions of organ procurement can be found in Table 2.

**Table 2:** Definitions of Legal and Ethical Considerations of Organ Procurement Citations: [18-20,22,23,32].

Term	Summary
Presumed Consent Model	Defined as permission to allow organ procurement from the donor unless the donor has explicitly stated that they do not want to be an organ donor. (Also referred to as an opt-out system.)
Donation Model	Consent must be obtained prior to the commencement of organ procurement. (Also referred to as an opt-in system.)
Uniform Anatomical Gift Act (UAGA)	A US federal law that governs how organ donation can be made. It permits the donation of organs for transplantation or medical research. This law permits a healthcare proxy of the deceased patient to consent to organ procurement.
Dead-Donor Rule	Organ procurement should not precipitate the death of the potential donor. The donor must be declared dead prior to the retrieval of organs.
Uniform Determination of Death Act (UDDA)	Defines death of the donor as the irreversible cessation of brain function or irreversible cessation of cardiopulmonary function.

Resource Management of Procurement in Operating Rooms and Systems of Care

In the US, the United Network for Organ Sharing (UNOS) establishes the framework for organ donation. UNOS is a private contractor which receives funding from the Center for Medicare and Medicaid Services (CMS) through Medicare. As of January 2023, UNOS oversees 56 regional organ procurement organizations (OPOs) nationwide. OPOs are responsible for coordinating the procurement of organs, obtaining consent for organ donation, providing support for donor families, and educating the public about organ donation with the goal of increasing donor consent rates. Organ procurement and distribution require complex coordination between retrieving and receiving institutions as well as donor and recipient surgeons, making OPOs essential. These organizations are closely involved in optimizing the donor and organ function, determining the suitability for transplantation, and preservation of the organs retrieved. Given the amount of coordination involved, organ procurement interventions cannot be performed without lead time or until an OR becomes available. They are often performed during off hours when OR utilization is at its nadir; timing, however, corresponds to a period of limited personnel resources. Procurement needs to be triaged with consideration, given that other urgent or semi-urgent cases may be competing for OR time. The anesthesiologist's role is essential in coordinating the effort since organ procurement may result in lifesaving transplants.

OPOs work closely to coordinate with regional procurement centers (RPCs). RPCs accept DBD donors from other regional (or area) hospitals with the purpose of organ procurement. The benefit of having RPCs is that other, often lower acuity hospitals, do not have to manage donors. This frees up their resources, such as critical care capacity, staffing, and OR time. In turn, the RPCs can provide better standardized and coordinated care. Statistics show that procurement at designated OPO centers provides a 27.5% increased organ yield per donor as compared to organ donation at a standard care facility [33]. RPCs are more cost-effective than standard care facilities [34]. In addition, CMS provides reimbursement to the center that performs organ procurement [34]. Overall, RPCs minimize the cost of procuring organs while providing a higher yield of organs and more standardized care [33,34].

Donation after brain death

Preoperative Role of the Anesthesiologist for Organ Procurement After Brain Death

It is the anesthesiologist’s role to be familiar with the process of determining brain death or death by neurologic criteria. According to the CMS, OPOs are responsible for verifying that a potential organ donor has met the criteria for brain death set by the AAN or the World Brain Death Project [35,36]. These standards state that in the absence of hypothermia and drug effects, a physical exam initiates the process of diagnosing brain death. Exam findings conclusive for brain death include a complete loss of consciousness, brainstem reflexes, and independent capacity for respiratory drive [10,37]. Ancillary tests can also be conducted if there are contradictory testing or exam findings. For example, apnea testing can examine the brain stem's ability to initiate a spontaneous respiratory effort in a state of hypercapnia confirmed by arterial blood pCO2 levels [38]. In addition, cerebral angiography, transcranial Doppler ultrasound, computed tomography angiography, and radionuclide brain imaging can be used to corroborate the diagnosis of brain death by establishing the lack of cerebral blood flow [38,39]. Practice deviations can exist between regional/area hospitals and may not always conform to national standards, which can lead to inaccurate diagnoses of brain death [35]. An accurate diagnosis of brain death is required so potential donors meet the legal criteria to become organ donors [31,32,35].

Anesthesiologists are responsible for preoperative donor assessment after donation consent is obtained [5]. This assessment includes reviewing the patient's hospital course, blood type, infectious disease status, and the etiology of death [5,40]. Routine laboratory tests are commonly ordered preoperatively, which include a basic metabolic panel, a complete blood count, coagulation studies, and evaluation of the acid-base status of the donor [5,40]. Before a donor is transferred to the operating room, the anesthesiologist must ensure that appropriate monitoring is in place and that electrolyte abnormalities are corrected. Blood pressure, temperature, pulse oxygen saturation, urine output, and heart rate/rhythm monitoring are standard monitors [8,41]. Noninvasive blood pressure is the most used hemodynamic variable. Arterial lines and central venous catheters can be placed for additional monitoring, but this varies among institutions [4]. Anesthesiologists should accompany the patient during transport to the operating room to manage unanticipated cardiovascular instability to preserve organ function and viability and limit warm ischemia [41]. Specific goals to optimize organ viability and transplant outcomes depend on which organs are being procured and will be discussed below. There are no national or international guidelines for intraoperative management of the brain-dead organ donor, but much can be learned from the guidelines published regarding ICU care of the brain-dead donor.

Physiologic Derangements and Intraoperative Considerations in Brain Death 

Cardiovascular: Prior to brain death, increased intracranial pressure can induce a “catecholamine surge” via activation of the sympathetic nervous system in response to decreased brain perfusion [4,41,42]. The catecholamine surge increases the donor's peripheral vascular resistance and heart rate, thereby increasing myocardial oxygen consumption and decreasing myocardial perfusion [43]. Myocardial injury preceding brain death may contribute to hypotension and rhythm disturbance. [44]. 20%-25% of brain-dead donors have evidence of myocardial ischemia, with 40% showing echocardiographic evidence of myocardial dysfunction [45]. There is some evidence that treatment with beta-blockers can mitigate a catecholamine surge, preventing a rise in systolic blood pressure and preserving left ventricular function [46,47]. According to the recommendations of the Society of Critical Care Medicine/American College of Chest Physicians/Association of Organ Procurement Organizations Consensus Statement, initial hypertension should be managed with esmolol infusions [4]. After a period of hypertension, donors usually develop hypotension secondary to the loss of vascular tone and fluid regulation.

There is significant variation in the recommendations for a donor’s hemodynamic parameter goals. In the United Kingdom, the National Health Service (NHS) guidelines recommend a mean arterial pressure (MAP) between 60 and 80 mmHg [48]. UNOS recommends that MAP be maintained between 60 and 100 mmHg, and Canadian guidelines recommend MAP >70 mmHg [7,49,50]. Most published guidelines state that achieving hemodynamic stability begins with volume resuscitation with either normal saline or a balanced salt solution [41]. Starch-based colloid solutions are generally contraindicated in volume resuscitation for DBD patients because of delayed graft functions in the recipient [41,51]. However, as an exception, if lung procurement is planned, a colloid is preferred to a crystalloid to maintain the hemodynamic stability of the donor [52]. 

Vasopressor and inotrope therapy can be used when hemodynamic stability cannot be achieved through volume resuscitation alone [5,8]. The choice of vasoactive/inotropic agent is dictated by the type of hemodynamic and cardiac rhythm disturbances seen in the donor. In a retrospective cohort study, 97.1% of donors received vasopressors for hemodynamic support [53]. There are conflicting data favoriting a specific vasoactive therapy. Dopamine was classically used in the ICU setting to support DBD donors but has been largely replaced by norepinephrine in many countries worldwide [54]. Norepinephrine is generally the preferred agent for hemodynamic support in Europe [48]. Vasopressin is recommended for use in other countries such as Canada, Ireland, and India. In addition, vasopressin has been recommended by the American College of Cardiology as a first-line treatment to restore adequate perfusion pressure for organ donation [43,50]. Vasopressin has advantages over inotropic drugs. Not only does it elevate blood pressure, but it also can treat diabetes insipidus in DBD donors with pituitary dysfunction [55,56]. Furthermore, vasopressin therapy has been associated with an increased rate of organ procurement [57-59].

After a marked period of sympathetic outflow, as previously discussed, bradycardia can occur in the DBD donor (Cushing’s Reflex). Bradycardia was seen in about one-third of DBD donors [60]. Dobutamine is recommended by the NHS and the American Society of Anesthesiologists for the treatment of bradycardia. The target heart rate for a DBD donor is between 60 and 120 beats per minute [48,61,62].

Respiratory: Perturbations of pulmonary function in the brain-dead donor are multifactorial and are mainly due to neurogenic pulmonary edema (NPE) [8,43]. NPE is characterized by an increase in pulmonary vascular resistance that causes increased hydrostatic pressure and transudative fluid extravasation into the interstitial and alveolar lung spaces. Alveolar edema impairs oxygenation in the donor due to ventilation/perfusion mismatch [41]. Treatment with beta-2 adrenergic agonists has been shown to reduce alveolar edema ex-vivo by increasing the alveolar fluid clearance rate [63-65]. However, a randomized trial on the effects of nebulized high-dose albuterol on oxygenation in DBD donors with pulmonary edema found that perioperative treatment with albuterol did not improve donor oxygenation and caused tachycardia. Treatment with high-dose albuterol should not be used to enhance the resolution of pulmonary edema to improve oxygenation in DBD patients [66].

During the period leading up to and throughout the procedure of organ donation, management of pulmonary dysfunction involves using lung protective ventilator settings in the DBD patient while ensuring optimal tissue oxygenation. The NHS recommends lung-protective ventilation with volumes of 4-8ml/kg ideal body weight, with optimal positive end-expiratory pressure between 5 and 10cm H₂O [41,63]. In addition to lung-protective ventilation settings, pulmonary recruitment maneuvers may be initiated to optimize oxygenation in the DBD donor. By increasing transpulmonary pressure, recruitment maneuvers expand collapsed alveoli leading to the improved matching of ventilation and perfusion [67].

Endocrine: Cessation of blood supply to the brain may disrupt the hypothalamic-pituitary axis (HPA). Disruption of the HPA decreases the serum concentrations of thyroid-stimulating hormone, adrenocorticotropic hormone, and antidiuretic hormone (ADH) [8,68-70]. The literature is conflicting on the benefit of hormone replacement therapy (HRT); however, current recommendations still include HRT with T3, T4, a corticosteroid, and ADH prior to organ procurement in all patients [71-74]. 

Thyroid hormone replacement is postulated to increase the total number of organs procured per donor by optimizing organ perfusion. This is presumably related to increased automaticity of the pacemaker cells in the myocardium and upregulating ATPase-rich alpha-heavy chains. Overall, this results in increased perfusion of the myocardium [75]. In a retrospective study, pretreating DBD patients with exogenous T3 and T4 increased the number of organs procured per donor by 12.8-15.3% compared to donors who were not given thyroid hormone replacement [72].

Corticosteroid therapy is administered in the DBD patient to provide hemodynamic stability via its beta-adrenergic activity on the vasculature. In DBD patients, corticosteroid therapy has increased lung recovery and reduced renal graft failure rates [76]. Therapy with corticosteroids decreased the need to use vasopressors by 20% in DBD patients as compared to patients who did not receive corticosteroid treatment [76]. Furthermore, the use of corticosteroids in DBD patients is believed to decrease the global inflammatory response observed after brain death. Observational studies demonstrate that corticosteroid therapy in DBD patients results in an increased total number of organs procured. Based on the results of these observational studies, further randomized controlled trials were conducted but did not demonstrate an increase in the total number of organs procured relative to donors with or without steroid therapy [77]. Despite the conflicting data, corticosteroid treatment is still generally used in practice [71].

Both brain death and corticosteroid treatment can lead to an increase in serum glucose levels [76]. In DBD patients, hyperglycemia can be worsened by the release of epinephrine, administration of exogenous steroids, or infusions of medications and solutions containing dextrose [41]. Therefore, it is generally recommended to maintain serum glucose levels below 180 mg/dL with insulin infusion therapy, as hyperglycemia after brain death is associated with poor transplanted organ function [76].

Brain death can also affect the posterior pituitary gland leading to decreased ADH release. As a result, the DBD patient can develop diabetes insipidus, which may lead to polyuria, hypovolemia, and elevated serum osmolarity [8]. Hormone replacement therapy (HRT) with arginine vasopressin (AVP) can treat diabetes insipidus and decrease serum osmolarity. Treatment with AVP resulted in increased successful recovery rates for organs and decreased post-transplant rejection, as previously discussed [76]. 

Thermoregulation and therapeutic hypothermia: In the DBD donor, temperature dysregulation occurs secondary to damage to the hypothalamus, precipitating hypothermia. Additionally, decreased metabolic rate in the donor patient leads to reduced heat production, potentiating the effect of temperature dysregulation [78]. Hypothermia is, therefore, commonly seen in the DBD donor. Hyperthermia is rare. There has been at least one case report of malignant hyperthermia in an organ donor [79]. Traditionally, the patient’s temperature has been maintained between 36.5 and 37.5 degrees Celsius. The desired temperature target can be achieved with forced air convection devices, fluid warmers for all intravenous infusions, and warming the OR before donor arrival [41].

Some studies suggest that mild hypothermia of the DBD donor improves renal graft function [80, 81]. A trial concluded that a donor temperature between 34 and 35 degrees Celsius reduced the frequency of delayed renal graft function in the recipient compared to normothermic donors [80]. More recent studies contradict these findings [82]. More studies are needed to assess the effect of mild donor hypothermia on graft function of other organs.

Hematologic: Coagulopathy has been reported in 10% to 80% of patients after brain death, and it is postulated to be due to the release of tissue factors from injured brain cells initiating the coagulation cascade and resulting in consumptive coagulopathy [53,83]. Coagulopathy is usually treated only when there is active bleeding, utilizing blood products [8]. Recommendations in Canada target hemoglobin concentration between 9 and 10 g*dL-1 [71]. Generally, blood products can be given as needed, particularly in instances with significant surgical bleeding, but this is not without risk of transfusion reactions and graft dysfunction [84,85]. In a retrospective study, however, blood transfusion in the donor was independently associated with a 23% decrease in the risk of delayed graft function in kidney transplant recipients [85]. Leuko-reduced blood is often preferred to eliminate the risk of acute transfusion reactions in the donor [84].

Musculoskeletal management*: *Recommendations for the perioperative management of a brain-dead donor include using neuromuscular blocking agents to abolish spinal reflexes [86,87]. Intact spinal reflexes mediate muscle movements during organ recovery and can interfere with surgical maneuvers [87]. The use of neuromuscular blocking agents inhibits the spinal reflexes that remain intact after brain death. A retrospective study from the Harborview Medical Center in Seattle found that their anesthesiology department used neuromuscular blocking agents in about 90% of organ procurement cases [7].

Inhalational anesthesia for organ procurement: While brain-dead patients have irreversible damage to the brain and brainstem, they can still respond to surgical stimuli via spinal reflex arcs. Stimulating spinal reflexes can mediate a cardiovascular response inducing a state of hypertension and increased catecholamine concentration [88-91]. Inhaled volatile anesthetics are initiated to alleviate the donor's hypertension. Volatile anesthetics here may be of additional benefit due to their proposed effect on the ischemic preconditioning of cardiac and hepatic tissue [42].

The use of inhalational anesthetics is postulated to have a protective effect on tissues, known as anesthetic preconditioning (APC). APC could cause changes at the biomolecular level that reduce adrenergic responses, leading to fewer instances of infiltration of pro-inflammatory substances such as TNF-α into procured organs. APC reduces ischemia-reperfusion injury and organ dysfunction in the heart, kidney, and liver [88]. Inhalational anesthetics could decrease the possibility of acute dysfunction of transplants and increase positive transplant outcomes [88,92-94].

A study demonstrated that inhaled sevoflurane anesthetic during organ recovery yielded a lower incidence of graft dysfunction in the recipient [90]. However, in another study, Perez-Protto et al. demonstrated that anesthetic preconditioning did not provide added benefits for either graft survival or function in the recipient [91]. Due to discrepant published findings, further research is required to determine the effect of volatile anesthetics on ischemic damage to organs during organ recovery.

Based on a survey in France and a single-center study by Lele et al., approximately 2/3 of DBD donors may receive a volatile agent in the operating room during organ recovery procedures [7,44]. If volatile anesthetics are used in organ procurement, anesthesiologists must ensure only a minimally effective end-tidal concentration of volatile anesthetic is administered. Higher doses of volatile anesthetics are associated with increased vasodilation and even increased colloid use to maintain systemic blood pressure [88]. 

Intraoperative goals and organ procurement protocol

The major goal is to ensure that the donor remains hemodynamically stable throughout the perioperative period, which ensures adequate tissue oxygenation and optimizes organ recovery and organ function after transplantation [5,7]. Protocols and guidelines for anesthesiologists are based on limited and conflicted outcome data and vary in practice. The Harborview Medical Center published an ICU protocol in conjunction with their regional OPO and performed a retrospective review of organ donation anesthetics. Notably, 90% of patients received a neuromuscular blocker, 63.3% received an inhaled anesthetic (sevoflurane or isoflurane), and 33.9% of patients received an opioid, with fentanyl most often administered [7]. Intraoperative targets were met as follows: MAP 70 mmHg (93%), normothermia (96%), normoglycemia (84%), urine output of 1-3 mL · kg-1 · hr-1 (61%), and lung-protective ventilation (58%) [7]. There are limited evidence-guided protocols for intraoperative goals that optimize graft function; more research is needed to provide uniform recommendations. A general summary of intraoperative goals is included in Table 3.

**Table 3:** Intraoperative Goals for Organ Procurement Citations: [4,7,41,45,50-52,56,64,65,67,71-74,76,80,81,83,84,86-88].

Organ System	Intraoperative Goals	Intervention(s)
Cardiovascular	MAP 60-80 mmHg, Systolic BP > 90 mmHg, HR 60-120 beats/min, CVP 4-11 mmHg	Esmolol to manage initial hypertension. Initial resuscitation with crystalloids. *If lung procurement is planned, a colloid is preferred* Vasoactive therapy if hemodynamic stability is not achieved with fluids.
Respiratory	Tidal Volume 4-8ml/Kg ideal body weight, Positive End- expiratory pressure (PEEP) 5-10 cm H2O, SPO2 >95 %, PaO2 > 80 mmHg	Lung protective setting with low tidal volume and high PEEP. Pulmonary recruitment maneuvers.
Renal	Urinary output > 1 mL · kg^-1^ · hr^-1^, Maintain electrolyte balance	Electrolyte repletion and volume expansion.
Endocrine	Blood glucose level <180 mg/dL	Insulin infusion to maintain normoglycemia. Hormonal replacement with T3, T4, corticosteroid, and vasopressin (ADH).
Thermoregulation	Maintain temperature between 35.8 C and 37.8 C	Forced air convection devices or fluid warmers for intravenous infusions.
Hematologic	Hemoglobin concentration between 9 and 10 g·dL^ -1^	Transfusion of blood products if the patient is actively bleeding. *Leukoreduced blood is preferred when available*
Musculoskeletal	Inhibition of spinal reflexes	Neuromuscular blocking agents.
Anesthetic	Manage spinal reflexes and hypertensive crisis	Inhaled anesthetic (sevoflurane or isoflurane) at a minimally effective end-tidal concentration.

Donation after circulatory death 

Circulatory death can be recognized by a clinical exam that reveals the absence of responsiveness, heart sounds, pulses, and breath sounds. Confirmatory variables such as ECG, arterial monitoring, or echocardiography are used to corroborate the diagnosis of death and ensure prompt diagnosis. An obligatory observation period, or “no touch,” is mandatory to ensure no spontaneous return of circulation occurs [4,15]. The "no touch" interval is variable and most often institution based; it can range from 2-5 minutes or more. If circulatory and respiratory efforts do not resume within this interval, the potential donor is declared dead in conformity with the UDDA definition of death [95].

Most patients considered for DCD will be in the intensive care setting and dependent on ventilation and circulatory life support. Some of these patients will have neurologic derangement but will not meet the criteria for brain death. End-of-life care should be the priority for these patients as they are still completing the dying process. The anesthesiologist is not involved in discussing the donation of organs with the patient’s next of kin; this is usually the role of the critical care team or OPO employee [96]. It is recommended that the continuity of care for patients should be provided by a critical care attending physician or their designee. This individual also holds the responsibility to decide when to withdraw life support [6, 95]. When that time comes, the primary care team should withdraw care [6]. This transfer of care should only be passed onto a physician who is qualified in end-of-life care and who has a pre-existing relationship with the patient [6,96]. According to the American Society of Anesthesiologists' statement on DCD, anesthesiologists staffing operating rooms (and not a part of a patient's primary/critical care team) should not be required to participate in either the withdrawal of care, including disconnecting the patient from a ventilator and terminally extubating, or, in the declaration of death [6]. In cases of lung procurement in which donors are known to have difficult airways, the anesthesiologist may be asked to reintubate after death and facilitate the donation process [6]. If the anesthesiologist is asked to perform reintubation, they should be informed by the OPO before surgery, and the OPO should explicitly state their requirements. For this reason, anesthesiologists are encouraged to be familiar with local OPO and DCD protocols at their institutions [6].

The Maastricht classification for donors after circulatory death

The Maastricht classification was promulgated in 1995 to simplify the categorization of DCD donors. It aims to clarify the ischemic insults to organs based on the timing and location of cardiopulmonary death. The Maastricht classification ultimately guides which organs are suitable for retrieval and transplantation [97]. Its most recent revision is known as the Belgian-modified classification of Maastricht for donors after circulatory death (Table 4) [98]. There are five Maastricht classes split between two categories: uncontrolled DCD (uDCD) and controlled DCD (cDCD) [99]. In the United States, most DCD donors are class III donors [95].

**Table 4:** Summary of Belgian Proposed Classification for Donors After Circulatory Death *Euthanasia is legal in certain countries. In the USA, euthanasia is illegal in all 50 states. Citations: [98, 99].

Maastricht Category	Organs Procured
Uncontrolled DCD	
Class 1.* Dead on Arrival: *Victims of sudden death (in or out of the hospital) who have not been resuscitated	Kidneys, lungs, and sometimes liver
Class 2. *Unsuccessful Resuscitation: *A patient who has a cardiac arrest (in or out of the hospital) in which cardiopulmonary resuscitation was administered and was unsuccessful	
Controlled DCD	
Class 3. *Awaiting Cardiac Arrest: *Patients in whom withdrawal of life-sustaining treatment is initiated as agreed on by the healthcare professional and healthcare proxy	Kidneys, lungs, liver, pancreas, and sometimes heart (only class 3)
Class 4. *Cardiac Arrest While Brain Dead:* DBD patients that have a cardiac arrest while undergoing an organ procurement procedure	
Class 5. *Euthanasia:* Patients that are granted medically assisted circulatory death*	

Physiologic derangements and perioperative intervention after circulatory death

There is a paucity of literature on physiologic derangement in DCD. Cardiopulmonary instability, such as hypotension and hypoxia, is seen after cardiac death, and only ischemic injury to the heart is well characterized [100,101]. In contrast to the physiologic derangement after neurologic death, DCD patients are not susceptible to significant variations in body temperature or hematologic perturbations due to less severe neurological dysfunction than DBD patients. The physiologic derangements of DCD are related to ischemic changes after withdrawal of life-sustaining treatment and reperfusion injury. Generally, organ procurement in DCD occurs rapidly, in contrast to DBD patients who are on life support for an extended period. Specific details of perioperative intervention are based on reports of clinical experience and not clinical trials; however, interventions are focused on limiting organ ischemia.

Warm ischemic time

Organ procurement is limited by warm ischemic time (WIT). WIT is the time from circulatory arrest until cold flush, storage, or reperfusion. The longer the duration of warm ischemia, the less likely organs are to be transplanted [40]. Patients with unexpected cardiac arrest in the out-of-hospital setting are especially at risk for long periods of WIT. The organs of patients with out-of-hospital cardiac arrest are infrequently retrieved in the US; however, in Europe, they are frequently procured and can have successful outcomes [102,103].

Reperfusion techniques and procurement of the heart, liver, and kidneys in DCD

Procurement of the heart has predominantly been from DBD donors. Recent advancements in organ perfusion technologies allow the heart to be retrieved from DCD patients. Heart procurement from DCD donors is limited to cDCD Maastricht class III donors who have severe neurological damage but do not meet the criteria for brain death [104]. Under these circumstances, there is an opportunity to retrieve the heart prior to significant myocardial ischemia and myocardial damage. Procurement is initiated after the donor is declared dead following the withdrawal of life-sustaining treatment (WLST). There are two techniques used to procure the heart after circulatory death: direct procurement and perfusion (DPP) or normothermic regional perfusion (NRP) [104,105]. DPP involves a sternotomy, cardiectomy, and connection of the heart graft to an ex-vivo perfusion machine (frequently referred to as “heart in a box”) or cold storage/flush until transplantation [104]. NRP involves a sternotomy, exclusion of cerebral vasculature and selective restoration of blood supply to the thoracoabdominal vasculature by venoarterial-ECMO. During NRP, the heart resumes electrical and pump functions and may be evaluated for transplantation by direct visualization and/or transesophageal echocardiography. By utilizing NRP, hearts are exposed to minimal WIT and are given time to recover from warm ischemia after WLST [105].

NRP in DCD donors is also used to procure organs other than the heart. The liver is particularly sensitive to WIT after cardiac death, but instances of liver graft dysfunction have decreased due to the initiation of NRP techniques during DCD [106,107]. A study found that three-year graft survival rates for livers procured from 78 DCD patients were 80.2% [103]. In addition, kidneys retrieved from DCD donors have higher rates of delayed graft function compared to kidneys procured from DBD donors, but they are less sensitive to WIT than the liver [107,108]. Studies show kidney procurement using NRP techniques leads to a lower incidence of primary non-functioning and delayed graft function in comparison to DCD donors managed without NRP [108,109]. A United Kingdom transplant center found the one-year graft loss rate for kidneys using NRP was 3.4% compared to 6%, where NRP was not utilized [110].

## Conclusions

Anesthesiologists play a significant role in the organ procurement process. Their role in the perioperative management of the donor may affect the outcomes of organ transplantation. The gap between the number of organs harvested and the number of patients awaiting organ transplantation remains high despite continued efforts to increase the number of available organs. A high transplantation success rate is crucial to ensure the best use of the scarce supply of organs. As members of the organ procurement team, anesthesiologists should be aware of ethical, legal, systems-based, and clinical considerations when providing anesthetic care to patients who are declared dead by neurologic and/or cardiopulmonary criteria. Furthermore, intraoperative management during organ procurement after brain death focuses on the associated unique physiologic derangements and targets the optimization of the viability of organs intended for procurement and transplantation. The optimal management of donors has been demonstrated to positively affect the clinical outcomes of transplantation and the success of organ recovery.
